# Exploring the role of bacterial virulence factors and host elements in septic arthritis: insights from animal models for innovative therapies

**DOI:** 10.3389/fmicb.2024.1356982

**Published:** 2024-02-12

**Authors:** Tao Jin

**Affiliations:** ^1^Department of Rheumatology and Inflammation Research, Institute of Medicine, Sahlgrenska Academy, University of Gothenburg, Gothenburg, Sweden; ^2^Department of Rheumatology, Sahlgrenska University Hospital, Gothenburg, Sweden

**Keywords:** septic arthritis, animal models, bacterial virulence, host factors, therapeutic strategies

## Abstract

Septic arthritis, characterized as one of the most aggressive joint diseases, is primarily attributed to *Staphylococcus aureus* (*S. aureus*) and often results from hematogenous dissemination. Even with prompt treatment, septic arthritis frequently inflicts irreversible joint damage, leading to sustained joint dysfunction in a significant proportion of patients. Despite the unsatisfactory outcomes, current therapeutic approaches for septic arthritis have remained stagnant for decades. In the clinical context, devising innovative strategies to mitigate joint damage necessitates a profound comprehension of the pivotal disease mechanisms. This entails unraveling how bacterial virulence factors interact with host elements to facilitate bacterial invasion into the joint and identifying the principal drivers of joint damage. Leveraging animal models of septic arthritis emerges as a potent tool to achieve these objectives. This review provides a comprehensive overview of the historical evolution and recent advancements in septic arthritis models. Additionally, we address practical considerations regarding experimental protocols. Furthermore, we delve into the utility of these animal models, such as their contribution to the discovery of novel bacterial virulence factors and host elements that play pivotal roles in the initiation and progression of septic arthritis. Finally, we summarize the latest developments in novel therapeutic strategies against septic arthritis, leveraging insights gained from these unique animal models.

## Introduction of septic arthritis

Septic arthritis is the most aggressive joint disease. Even after receiving immediate treatment, the joint damage caused by septic arthritis is often irreversible, leading to permanent joint dysfunction in up to half of the patients (Kaandorp et al., 1995). Furthermore, the emergence of methicillin-resistant *Staphylococcus aureus* (MRSA) has severely complicated the available treatment options (DeLeo et al., 2010).

The yearly incidence of septic arthritis varies between 2 and 10 per 100,000 people in western countries (Morgan et al., 1996; Kaandorp et al., 1997; Weston et al., 1999; Geirsson et al., 2008). Due to increased orthopedic-related infections and an aging population, as well as enhanced use of immunosuppressive therapies and invasive procedures, the incidence of septic arthritis is rising over time (Mathews et al., 2010).

Several studies have consistently shown that *S. aureus* is the predominant causative organism, accounting for 40–54% of cases across all age and risk groups. Following closely are streptococci, another Gram-positive bacteria, contributing to 18–28% of cases. The third group comprises Gram-negative bacilli, such as *Haemophilus influenzae*, *Escherichia coli*, and *Pseudomonas aeruginosa*, constituting 15–19% of cases (Kaandorp et al., 1997; Ryan et al., 1997; Weston et al., 1999). Despite the rapid advancements in modern medicine, the distribution and antibiotic susceptibility profiles of pathogens causing septic arthritis have shown little change over time, as demonstrated in two studies (Dubost et al., 2002, 2014). Septic arthritis induced by Gram-negative bacilli is frequently observed in patients with co-morbid conditions like intravenous drug abuse and preexisting joint disorders (Smith et al., 2006). Intriguingly, it is noted that septic arthritis caused by Gram-negative bacilli is linked to a less favorable prognosis compared to cases caused by Gram-positive bacteria, exhibiting a higher mortality rate and greater joint dysfunction (Goldenberg et al., 1974; Bayer et al., 1977).

The most common route of septic arthritis is hematogenous spread of bacteria to the distant joints such as knees and shoulders (Goldenberg and Reed, 1985). The risk of septic arthritis caused by intra-articular injection of corticosteroids is very low, accounting for 1 in 12,000 injections (Weitoft and Makitalo, 1999). It is recognized that acute hematogenous osteomyelitis is particularly prevalent in younger children, typically under 5 years of age. This type of osteomyelitis commonly affects the metaphysis due to the rich but slow blood flow inherent in the growing bone (Iliadis and Ramachandran, 2017). Well-known risk factors for septic arthritis include aging, diabetes, hemodialysis, and intravenous drug abuse (Kaandorp et al., 1995; Al-Nammari et al., 2008). Interestingly, rheumatoid arthritis increases the risk of septic arthritis by 4–15 times (Doran et al., 2002). It has also been shown that modern biologics such as TNF inhibitors may increase the risk of septic arthritis in rheumatoid arthritis (RA) patients compared to patients receiving conventional disease-modifying antirheumatic drugs (DMARDs; Galloway et al., 2011).

The typical clinical symptoms of septic arthritis include monoarthritis in the large joints. Knees are the most commonly affected joints, accounting for over 50% of septic arthritis cases. Some patients may also experience oligo or polyarthritis, which is associated with a significantly higher mortality rate. Blood cultures yield positive results in approximately 50% of septic arthritis patients, while synovial fluid cultures show positive results in around 80% of cases (Mathews et al., 2010). A comparative analysis investigated various culture systems for synovial fluid samples from horses with septic arthritis. The findings revealed that blood culture medium enrichment outperformed other techniques, such as direct agar culture, agar culture following lysis-centrifugation pretreatment, and agar culture after conventional enrichment (Dumoulin et al., 2010). Nevertheless, a study involving 90 adult patients with acute knee joint arthritis suggested that the choice of culture method may be less crucial (Kortekangas et al., 1995). In some instances, synovial fluid cultures may be negative due to the collection of fluid after initiating antibiotic treatment.

The management of septic arthritis involves immediate initiation of antibiotic treatment upon suspicion of the condition. Additionally, the removal of intra-articular pus through repeated closed-needle aspiration and surgical aspiration via arthroscopy is crucial (Mathews et al., 2010). The significance of pus removal lies in the fact that our research has demonstrated the highly arthritogenic nature of antibiotic-killed *S. aureus* and certain bacterial components (Ali et al., 2015c; Mohammad et al., 2019). However, despite prompt treatment, the outcomes are often unsatisfactory, as septic arthritis frequently leads to irreversible joint damage. Up to half of the patients experience permanent joint dysfunction (Kaandorp et al., 1995).

Progress in the development of new treatments for septic arthritis has been stagnant. The current treatment options remain unchanged for the past 30 years. Therefore, elucidating the molecular mechanisms underlying the interplay between host factors and bacteria in disease development would represent a significant breakthrough in understanding septic arthritis etiology. It could potentially open avenues for novel therapeutic modalities, such as *S. aureus* vaccination, to combat this devastating disease. To achieve these objectives, it is crucial to establish a validated animal model that closely mimics the human disease.

## Key questions in septic arthritis

**Figure 1:**
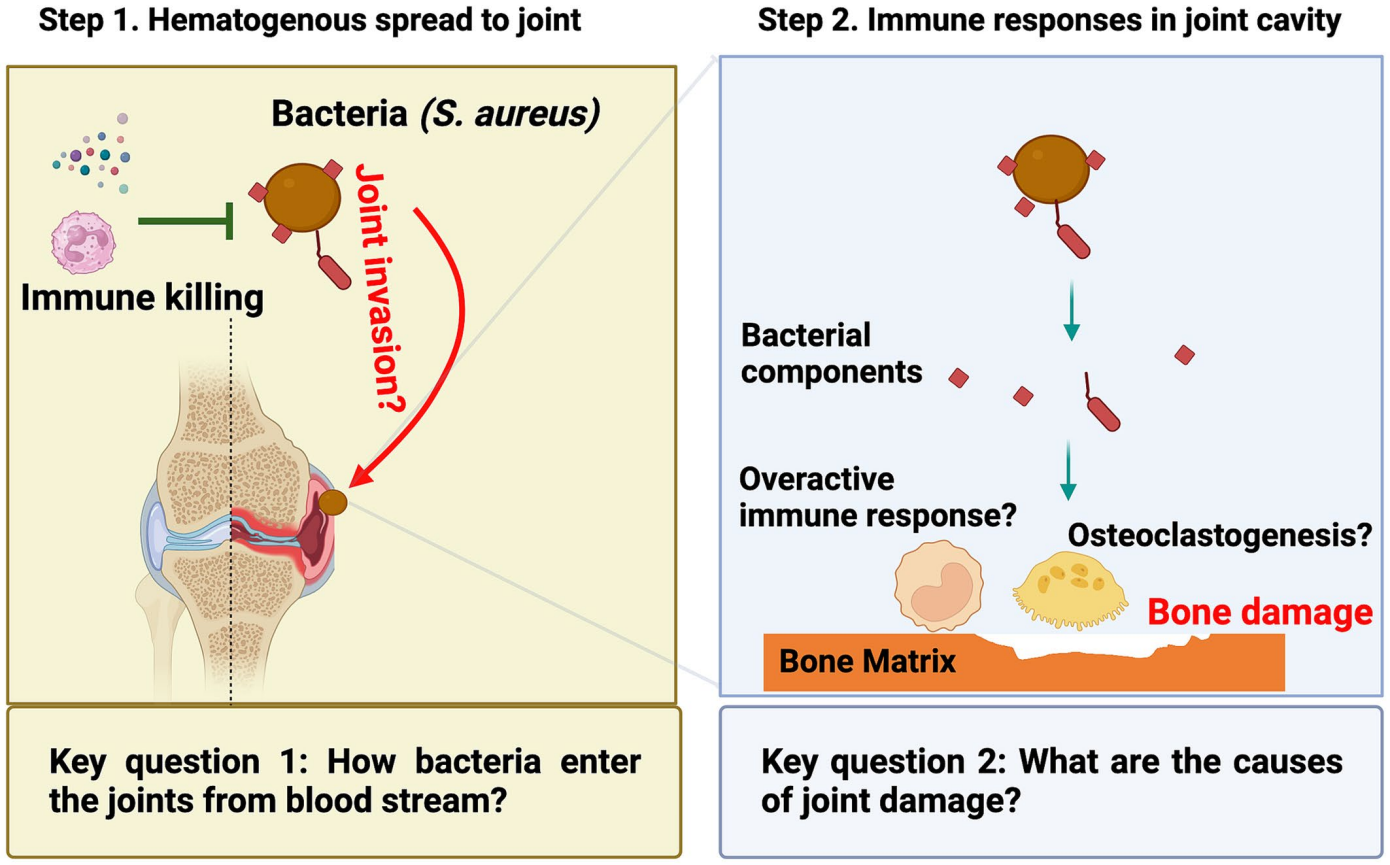
In septic arthritis research, two unresolved scientific questions persist: (1) During the initial phase when bacteria are in the bloodstream, the mechanism by which they access the joint cavity to induce the disease remains unclear; (2) In the later phase when bacteria have reached the joint, the primary cause of joint damage remains an unresolved inquiry.

There are two critical questions related to the disease’s development (Figure 1). The first question is how bacteria enter the joint cavity from the bloodstream. The majority of septic arthritis cases result from hematogenous spreading. Bacteria can enter the bloodstream through various routes, including wounds, surgical procedures, or dental treatments and procedures. The heightened bacterial survival in the bloodstream significantly elevates the probability of these bacteria reaching distant joints and triggering septic arthritis. This is correlated with an augmented bacterial load in organs, notably the kidneys. At this stage, the innate immunity becomes pivotal in eradicating bacteria from the bloodstream, a topic to be explored in detail in the later chapter dedicated to the role of host factors in septic arthritis. Following survival from innate immune defenses, bacteria utilize adhesins, such as *S. aureus* surface proteins, to play a pivotal role in invading joints. These adhesins adhere to components of the extracellular matrix or joints, ultimately leading to the development of septic arthritis. It is important to note that in this context, bacterial adhesins generally play a limited role in protecting bacteria from immune eradication and may not markedly impact the bacterial load in kidneys. A comprehensive understanding of the molecular mechanisms underlying bacterial joint invasion will ultimately pave the way for the development of prophylactic strategies against septic arthritis.

The second question concerns the underlying cause of joint damage, which occurs as a consequence of bone erosion after the bacteria reach the joint. Bone erosion in septic arthritis can occur rapidly and be very severe (Fatima et al., 2017). It was believed to be caused by a combination of bacterial toxins, host immune response, and consequent tissue damage. In recent years, it has become increasingly evident that bacterial components, including *S. aureus* lipoproteins, exhibit robust arthritogenic properties leading to bone erosion. Importantly, a growing body of evidence indicates that the swift focal bone destruction is triggered by the activation of local osteoclastogenesis in septic arthritis. Understanding the underlying mechanisms of joint damage could help identify new targets for therapeutic interventions to prevent or reduce joint damage in patients with septic arthritis.

## The model system of septic arthritis

**Figure 2:**
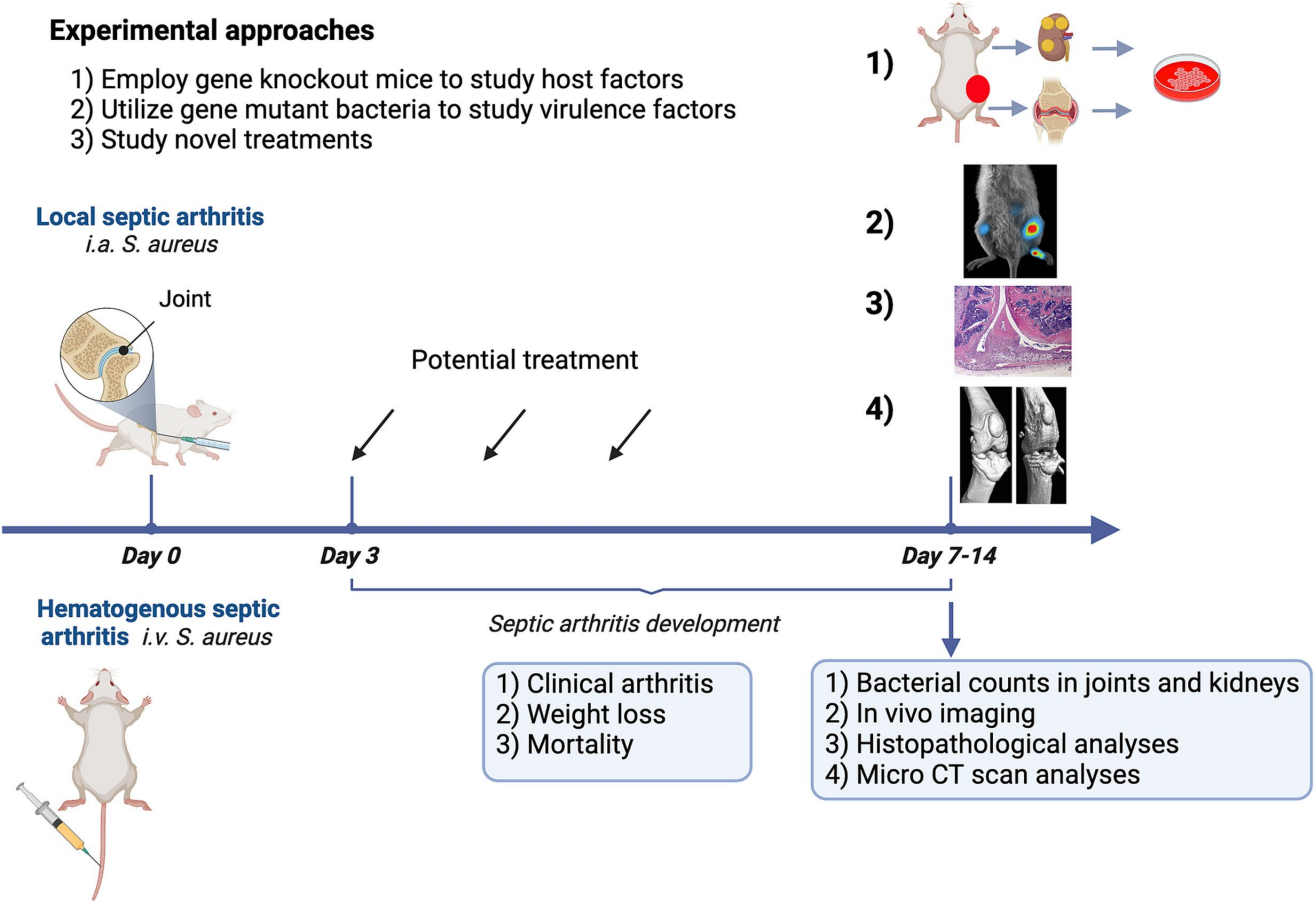
Experimental approaches employing a mouse model for septic arthritis aim to investigate the role of host factors, identify bacterial virulence factors, and design novel therapeutic strategies.

There are two models for septic arthritis: hematogenous septic arthritis and local septic arthritis models (Figure 2). We propose that the development of hematogenous septic arthritis should be divided into two stages—early and late. During the early stage of infection, bacteria need to survive the bactericidal components and phagocyte attacks in the blood, to disseminate to synovial tissue, and finally to reach the joint cavity and initiate the disease. In the joint cavity (late stage), bacteria proliferate and release a vast arsenal of components that arouse a host response and cause joint damage. The hematogenous model closely mirrors the clinical scenario experienced by the majority of patients, in contrast to the local model involving intra-articular bacterial inoculation. However, the local septic arthritis model serves as a valuable tool for studying later-stage immune responses *in situ*, as the intra-articular route bypasses the early phase of disease pathogenesis. Notably, the predominant body of research in this field has been conducted in rodents, with a primary focus on murine models. This review delves into the history, practical considerations, and recent advances concerning these two models in mice. Additionally, this section concludes with a concise overview of septic arthritis models in large animals, as well as a discussion on animal models for osteomyelitis.

### History of the animal models for septic arthritis

Professor Tarkowski at Sahlgrenska University Hospital in Gothenburg, Sweden pioneered the development of an animal model system for septic arthritis, utilizing it to investigate bacterial virulence factors and immune responses associated with this condition. In the 1990s, Professor Tarkowski and his colleagues including Dr. Bremell serendipitously observed a spontaneous outbreak of septic arthritis in mice within their animal facility at Mikrobiologen, Gothenburg University, Sweden (Bremell et al., 1990). They successfully isolated the bacteria responsible for the joint infections and identified it as *S. aureus*. To honor the laboratory technician, Lena Svensson, who played a crucial role in identification of *S. aureus* in mice with spontaneous outbreak of septic arthritis, they named the isolated strain LS-1. Extensive studies confirmed that the LS-1 strain exhibited significant arthritogenic properties, and consequently, it became widely employed in subsequent animal models of septic arthritis (Bremell et al., 1991). It is however important to note the crucial origin of the LS-1 strain, as the clinical significance of using LS-1 may be compromised if it significantly diverges from well-established strains like Newman, derived from a human infection in 1952 (Duthie and Lorenz, 1952). Although LS-1’s genome sequencing is pending, it shares numerous known virulence factors with the Newman strain, such as clumping factors and protein A, while lacking staphylokinase. A study deleting clumping factor A in both Newman and LS-1 revealed similar clinical outcomes in mice (Josefsson et al., 2008).

### Practical issues with the mouse model for septic arthritis

As *S. aureus* remains the primary cause of septic arthritis, it is commonly employed in related studies. Nevertheless, various bacterial species, such as *Streptococcus pyogenes*, have been shown to induce septic arthritis in mice (Sakurai et al., 2003). Furthermore, *Pseudomonas aeruginosa*, a gram-negative bacteria, has been demonstrated to induce septic arthritis in a comparable hematogenous mouse model, albeit with lower frequency and higher mortality (Jin et al., 2019). Mice are commonly used due to their cost-effectiveness and the availability of genetically modified strains. However, it is worth noting that rats have also been demonstrated to be sensitive to arthritogenic strains of *S. aureus* (Bremell et al., 1994; Gillett et al., 2010). In practice, most of experimental mice, including NMRI, C57BL6, and BALB/C mice, can be used in this model system. However, the optimal arthritogenic dose may vary among mouse strains, so it is important to titrate the dose before starting the experiments. Generally, C57BL6 mice are more resistant to infection compared to NMRI and BALB/C mice, and they require a higher dose to induce septic arthritis. Since C57BL6 mice are widely available and many genetically modified mice are derived from this strain, they are often used in studies aiming to understand the role of host factors, along with different gene knockout mice. On the other hand, for studies testing the effects of treatments, we usually choose NMRI mice, which are an outbred mouse strain with larger genetic variations, resembling the clinical situation.

Septic arthritis models can serve various purposes, including: (1) examining host factors through the utilization of genetically modified mice; (2) exploring virulence factors using gene mutant bacterial strains; and (3) advancing the development of novel treatments. Figure 2 provides a summary of the experimental approaches. *Staphylococcus aureus* will be intravenously inoculated through the tail vein of mice at an arthritogenic dose. The optimal arthritogenic dose varies among strains. The most arthritogenic strain we have tested is the Newman strain, which has been used to create genetically modified strains. The LS-1 strain is also highly useful for studying the development of septic arthritis in mice. However, the AB-1 strain is less arthritogenic but leads to high mortality. For instance, an intravenous injection of 2−5 × 10^6^ CFU/mouse in 200 μL of PBS with the Newman strain will result in septic arthritis development on days 2–3 post-infection. Both frequency and severity of clinical arthritis continues to rise until day 7 post-infection and then stabilizes until day 10–14 post-infection.

Weight loss is another important clinical parameter that reflects the severity of the disease. Typically, mice do not experience weight loss in the first 1–2 days. From day 3 onwards, they start to lose weight, which continues until day 7. Most mice stabilize in terms of weight development from day 7, and some of them even start to gain weight. Mice with more severe disease will continue to lose weight. There will always be some mortality, ranging from 10 to 30% in this model. Interestingly, mice that die at very early time points (before day 3 post-infection) usually do not exhibit signs of severe infection and often die suddenly. Conversely, mice that die at later time points (after day 7 post-infection) usually continue to lose weight, and autopsy often reveals severe kidney abscesses. The severity and frequency of arthritis are often recorded daily. Since the frequency of arthritis does not increase after day 7 post-infection, we typically choose to conclude the experiment on day 10 or 14 post-infection. Kidney and liver samples will be collected to measure bacterial colony-forming units (CFU). Additionally, all joints, including deeper joints such as the shoulder, hip, and knee, which cannot be assessed clinically, will be collected for μCT scanning and later histopathological assessment.

In the local septic arthritis model, we typically inject live bacteria or bacterial components like *S. aureus* lipoproteins, TSST-1, and peptidoglycan into the knee joints of mice. It is crucial to have experienced researchers or technicians perform the joint injection procedure due to the small size of the mouse knees and the relatively challenging injection technique. The bacterial dose can range from 1 × 10^3^ to 1 × 10^5^ CFU/knee. We monitor the severity of joint inflammation by measuring the size of the knee. The experiment usually lasts 10–21 days, as bone erosions become apparent around days 7–10 after injection. Since the infection is localized, the animals generally do not experience significant weight loss. Unless immunosuppressive treatments are applied, no deaths should occur. At the end of the experiments, we collect blood and joints. Both knees are typically injected. One knee is homogenized to determine bacterial CFU counts and measure joint cytokine levels, while the lateral knees are preserved in formalin for future CT scanning and histopathological staining (Mohammad et al., 2019).

### New advancements

We have developed μCT methods to assess the severity of joint destruction in septic arthritis (Fatima et al., 2017). While histopathology is the gold standard for evaluating joint inflammation and destruction, it is a time-consuming and labor-intensive process. Therefore, we have been transitioning to modern μCT techniques in our laboratory for histopathological analyses. Currently, we are validating the use of an *in vivo* imaging system (IVIS) to quickly identify septic arthritis joints in our model (Deshmukh et al., 2023). It appears that this new technology is better suited for the local septic arthritis model rather than the hematogenous one. This distinction arises because the hematogenous spread of bacteria can reach the soft tissues and muscles near the joints, leading to abscess formation. These abscesses produce strong positive signals that can mislead the diagnosis of septic arthritis. However, the IVIS method is excellent for estimating bacterial load in a well-defined organ, such as the kidneys. The luminescent signals from kidney abscesses correlate perfectly with the CFU counts obtained from kidney homogenates (Deshmukh et al., 2023).

### Septic arthritis model in large animals

The mouse model presents numerous advantages, including the availability of a large number of genetically modified animals, cost-effectiveness, feasibility, and ethical considerations. However, there are limitations to the rodent model for septic arthritis. Rodents exhibit differences in cartilage biology, such as cartilage thickness and overall immune response, which are less similar to humans compared to large animals. Additionally, continuous monitoring of disease progression is challenging in rodents due to constraints in obtaining blood and larger volumes of synovial fluid from infected joints.

As a result, septic arthritis research has extended to large animals, including porcine (Zhao et al., 2023) and equines (Gilbertie et al., 2022), especially in endeavors to develop novel therapies. In large animals, septic arthritis is typically induced by intra-articular injection of bacteria. This approach is favored for its ease of execution, and the model system provides a more straightforward and stable readout compared to the hematogenous model. The joints chosen for injection are typically femorotibial joints in pigs and tibiotarsal joints in horses, with a bacterial dose usually set at 1 × 10^6^ CFU/joint (Gilbertie et al., 2022; Zhao et al., 2023). Bacterial components such as LPS have also been employed (Koziy et al., 2019).

The clinical course of the disease in large animals can be comprehensively evaluated through continuous assessments, including animal behavior measured by pain scores, bacteria counts in synovial fluids or synovial tissue, local joint inflammation assessed by ultrasound, proinflammatory biomarkers in blood and synovial fluids, as well as radiological and histological analyses of infected joints (Koziy et al., 2019; Gilbertie et al., 2022).

### Animal models for osteomyelitis

Peri-prosthetic joint infection (PJI) stands as a distinctive clinical entity, marked by significant differences from infections involving native joints, rendering it challenging to treat. The pathogenesis of PJI involves four key mechanisms: intracellular infections of bone cells, bacterial invasion of the osteocyte lacuna canalicular network in cortical bone, biofilm formation, and abscess formation (Masters et al., 2022). Numerous animal models contribute to our understanding of osteomyelitis and the advancement of treatment algorithms. These models range from simpler setups, where metal implants are placed under the skin or into cortical bone (Tshefu et al., 1983; Li et al., 2008), to more intricate models mimicking orthopedic devices (Arens et al., 2015). Additionally, there are models involving direct bacterial inoculation into vertebral bodies or intervertebral disks to induce vertebral osteomyelitis (Joyce et al., 2021). Noteworthy among them is a diabetic foot infection model, which involves bacterial inoculation into the footpad of diabetic obese mice (Farnsworth et al., 2015).

## To study the role of bacterial virulence factors in septic arthritis

**Figure 3:**
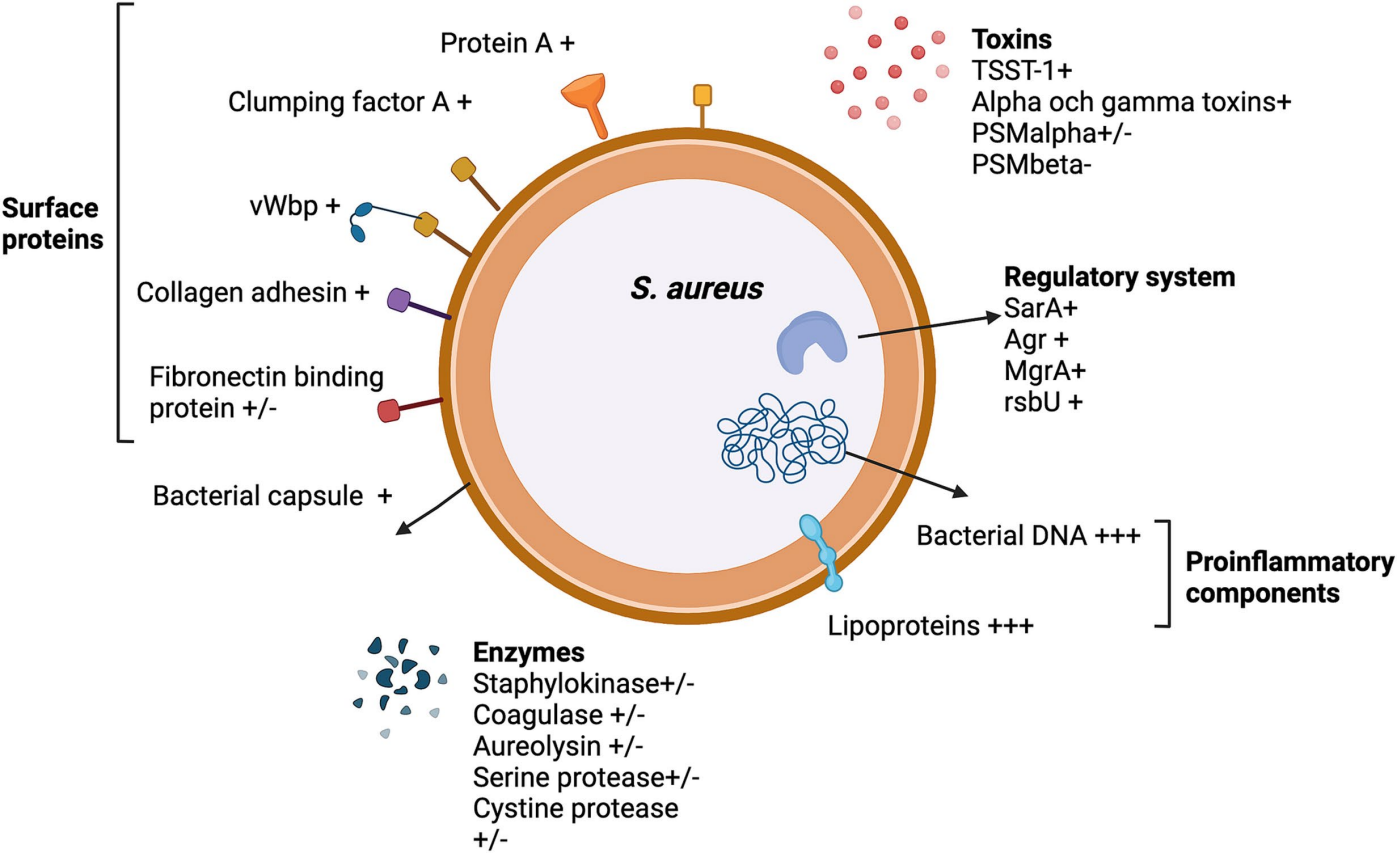
The *Staphylococcus aureus* virulence factors that impact septic arthritis pathogenesis. +, enhance septic arthritis; +/−, neutral effect; −, attenuate septic arthritis; and +++, arthritogenic effect by itself.

Bacteria produce a range of products that mediate tissue adhesion, facilitating the seeding procedure. Once an infection is established, other components are actively or passively released through the death and degradation of bacteria. Some of these components are highly proinflammatory and can induce strong inflammation in the joints, ultimately contributing to joint destruction (Jin et al., 2021). As *S. aureus* stands out as the primary causative agent of septic arthritis, research efforts to unravel the role of bacterial virulence factors have predominantly concentrated on this bacterial species. Figure 3 provides a summary of the *S. aureus* components implicated in septic arthritis. The majority of these studies have been conducted using the mouse model for hematogenous septic arthritis, with the exception of studies specifically addressing the arthritogenic properties of bacterial components, such as *S. aureus* lipoproteins, peptidoglycan, and bacterial DNA.

It is crucial to consider the presence of human-specific virulence factors, such as Panton-Valentine leukocidin (PVL), staphylococcal superantigen-like proteins, staphylokinase, and staphylococcal complement inhibitor (SCIN). These factors may necessitate non-physiological concentrations in mouse models for effective action in disease pathogenesis or could be ineffective altogether (Langley et al., 2005; Rooijakkers et al., 2005; Loffler et al., 2010; Kwiecinski et al., 2013). Therefore, validating mouse study results in a human setting or a humanized system becomes essential.

### Bacterial surface proteins mediating joint-specific invasion

Bacterial joint invasion stands as a pivotal step in the pathogenesis of septic arthritis, as the initial trigger for this ailment lies in the infiltration of *S. aureus* into the afflicted joints. The most frequently reported pathway for septic arthritis acquisition is the hematogenous spread of *S. aureus* to the synovial membrane of the joints (Mathews et al., 2010). It is evident that *S. aureus* surface proteins assume a critical role in the invasion of bacteria into the joints, thereby causing septic arthritis. Notably, *S. aureus* lacking the sortase enzyme A that recognizes specific surface protein sorting signals, completely loses its arthritogenic capacity (Nilsson et al., 1997; Jonsson et al., 2003). Subsequent investigations into individual surface proteins have revealed that the expression of certain proteins, such as protein A (Palmqvist et al., 2002), clumping factor A (Palmqvist et al., 2005), and collagen adhesin (Patti et al., 1994), are crucial for the induction of septic arthritis. Conversely, some surface proteins, such as fibronectin-binding proteins, have shown no impact on the induction of septic arthritis (Palmqvist et al., 2005). Interestingly, the fibrinogen-binding adhesin has also been identified as a virulence factor for septic arthritis caused by another Gram-positive bacterium, *Streptococcus agalactiae* (Jonsson et al., 2005).

Among these surface proteins, clumping factor A has been the subject of intense study. The expression of clumping factor impedes macrophage phagocytosis (Palmqvist et al., 2004). Interestingly, the effect of clumping factor on septic arthritis does not appear to depend on the presence of fibrinogen, as treatment with ancrod, a defibrinogenating enzyme, did not alter the course of septic arthritis induced by ClfA-expressing *S. aureus* (Palmqvist et al., 2004). In another study, mutations in the fibrinogen binding sites P336 and Y338 of clumping factor A nearly abolish *S. aureus*’s capacity to induce septic arthritis, underscoring the crucial role of fibrinogen binding activity in provoking septic arthritis (Josefsson et al., 2008).

**Figure 4:**
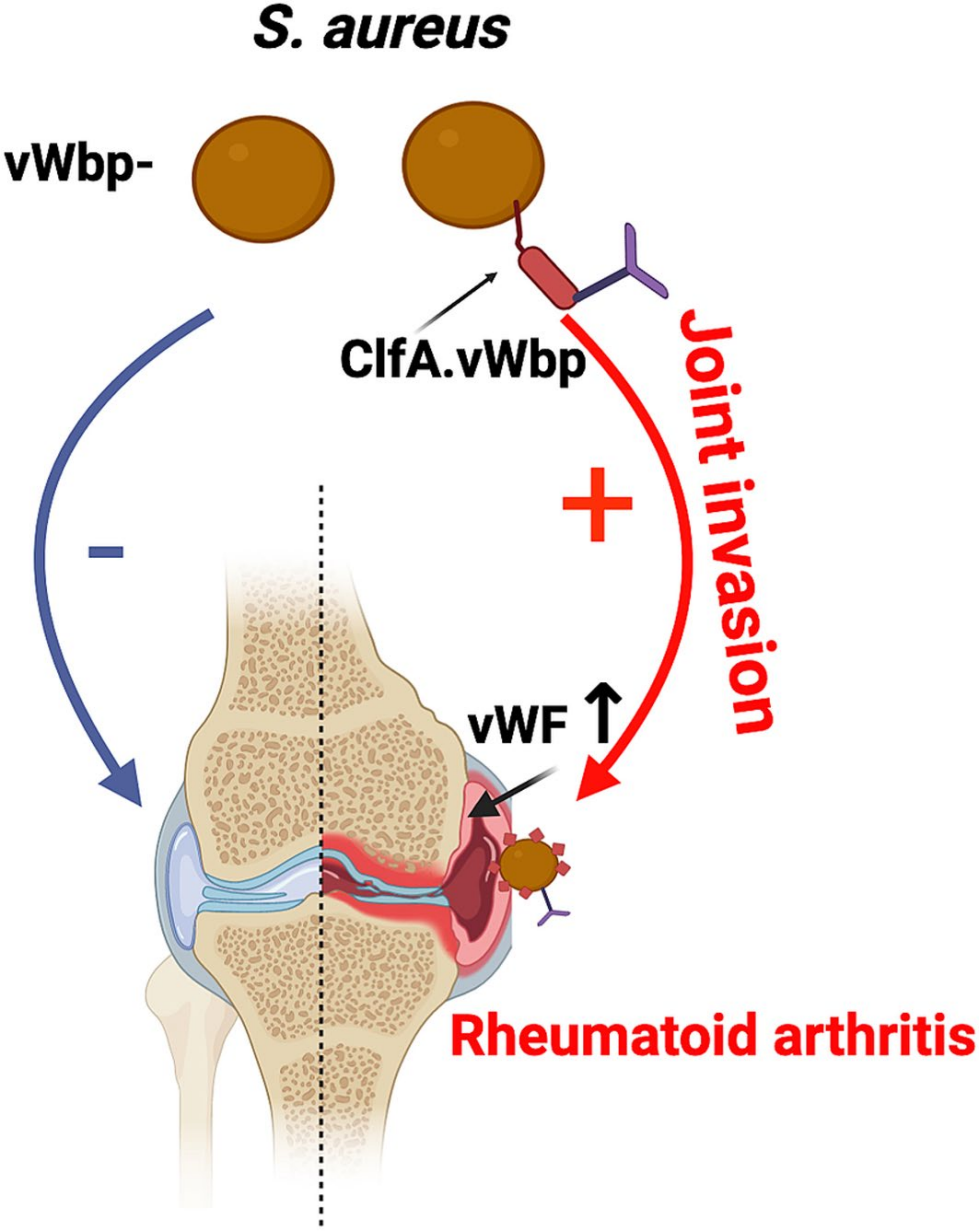
The hypothesis of a potential mechanism for the heightened susceptibility of rheumatoid arthritis patients to septic arthritis. The interaction between Clumping factor A (ClfA), von Willebrand binding protein (vWbp), and von Willebrand factor (vWF) mediates bacterial joint invasion, which may contribute to the increased vulnerability of RA patients to septic arthritis.

*Staphylococcus aureus* von Willebrand binding protein (vWbp) is a secreted protein, but it can anchor to the bacterial cell wall by binding to Clumping factor A (ClfA) (Claes et al., 2017). Notably, a recent report has suggested that ClfA, vWbp, and von Willebrand factor (vWF) form a robust complex, and that vWbp activates a direct, ultra-strong interaction between ClfA and vWF (Viljoen et al., 2021). Recent findings indicate that the depletion of vWbp in *S. aureus* eliminates the bacterium’s ability to invade the joint cavity, and this effect is mediated by an interaction between bacterial vWbp and host vWF (Na et al., 2020). Epidemiological studies have identified several risk factors for septic arthritis and RA emerges as one of the most significant factors, associated with a more than 10-fold higher risk (Favero et al., 2008). It is well-established that the level of vWF is increased in inflamed joints in RA (Blann, 1991). Inflammatory cytokines upregulate vWF release and inhibit the cleavage of vWF by suppressing the expression of ADAMTS13, an enzyme that cleaves vWF into smaller, less-active multimers (Bernardo et al., 2004). vWF forms ultra-large multimers anchored to the endothelial cell surface through clustered P-selectin upon endothelial cell activation (Padilla et al., 2004). These ultra-large vWF structures are known to mediate vascular adhesion of pathogens and significantly contribute to the onset of *S. aureus* endocarditis and cerebral malaria (Claes et al., 2014). Our hypothesis posits that inflammatory joint disease upregulates vWF levels, thereby enhancing the joint-specific invasiveness of bacteria (Figure 4). In the future, there is promising potential for designing therapeutic and prophylactic interventions targeting these molecules to prevent bacterial invasion of the joints.

Prosthetic joint infections pose a significant medical challenge, primarily due to the formation of antibiotic-resistant biofilms. Various lines of evidence suggest that surface proteins play a crucial role in promoting bacterial adhesion to extracellular matrix components, thereby contributing significantly to biofilm formation on indwelling medical devices (Foster, 2019). Studies have demonstrated that bacterial fibronectin- and fibrinogen-binding proteins play a pivotal role in the formation of macroscopic bacterial clumps in synovial fluids. Notably, pre-treating synovial fluids with plasmin or coating with tissue plasminogen activator has shown to reduce bacterial aggregate or biofilm formation, enhancing susceptibility to antibiotics (Kwiecinski et al., 2015; Gilbertie et al., 2019). Clinical investigations comparing *S. aureus* strains isolated from cardiac device colonization in human patients with those from uncomplicated bacteremia cases revealed that polymorphisms in the fibrinogen-binding protein A of *S. aureus* are associated with cardiac device infections. This suggests a propensity for *S. aureus* strains with high fibronectin affinity to adhere to indwelling devices in patients (Lower et al., 2011). Similar findings were observed, indicating that ClfA and fibronectin binding proteins mediate bacterial infection of implanted murine intra-aortic polyurethane patches (Arrecubieta et al., 2006). Recently, a mouse model of hematogenous implant infection was developed using orthopedic titanium implants placed in mouse legs. The surgical procedure with implant placement demonstrated a marked susceptibility to hematogenous implant-related infections compared to sham-operated legs. Notably, ClfA was identified as a pathogenic factor in *S. aureus* hematogenous implant-related infections, as neutralizing antibodies against ClfA inhibited biofilm formation *in vitro* and hematogenous implant-related infections *in vivo* (Wang et al., 2017).

In the context of osteomyelitis, a recent study demonstrated that the cell wall synthesis machinery can modulate *S. aureus* pathogenesis in osteomyelitis. This finding is supported by a significant reduction in septic implant loosening and *S. aureus* abscess formation within the medullary cavity, observed when utilizing penicillin-binding protein 3 or autolysin mutant strains in a mouse model for implant-associated osteomyelitis. Notably, *S. aureus* surface proteins C and ClfA seem to be dispensable in this particular context (Masters et al., 2021).

### Bacterial components inducing joint inflammation and destruction

Studies aimed at elucidating the arthritogenic properties of bacterial components often opt for the local septic arthritis model, involving the direct injection of bacterial components into the mouse knees. Antibiotic-killed *S. aureus* is arthritogenic and causes the joint destruction. This may explain the fact that permanent reduction in joint function is a common postinfectious complication in patients with septic arthritis (Ali et al., 2015c). *Staphylococcus aureus* lipoproteins play distinct roles in various infectious disease animal models (Mohammad et al., 2022). It is clear that *S. aureus* lipoproteins are extremely arthritogenic compared to other bacterial components that we have tested, as a single injection of *S. aureus* lipoproteins in mouse knees induced chronic destructive macroscopic arthritis that resembles the clinical septic arthritis (Mohammad et al., 2019). However, the expression of *S. aureus* lipoproteins has only a very limited effect on the induction and radiological bone erosion in hematogenous septic arthritis. This is evident as a *S. aureus* strain deficient in prelipoprotein lipidation resulted in similar severity of septic arthritis and bone destruction, as observed through micro-CT, compared to its parental strain (Mohammad et al., 2020). Interestingly, the *S. aureus* extracellular vesicles are proinflammatory and such proinflammatory properties are also dependent on the presence of *S. aureus* lipoproteins in extracellular vesicles (Kopparapu et al., 2021). It is known that *S. aureus* septic arthritis leads to a very quick bone resorption (Verdrengh et al., 2006). *Staphylococcus aureus* lipoproteins were shown to significantly contribute to bone resorption in *S. aureus* local septic arthritis and such effect is mediated by their lipid moiety through monocytes and macrophages (Schultz et al., 2022). Importantly, staphylococcal lipoproteins can trigger a shift toward fermentative metabolism in bone marrow-derived macrophages. Specifically, di-acylated lipoproteins more profoundly drive the metabolic shift in leukocytes, leading to increased joint inflammation and bone destruction *in vivo*, affirming the hypothesized connection between bacterial lipoprotein exposure, macrophage metabolic changes, and subsequent bone damage (Nguyen et al., 2023). *Staphylococcus aureus* peptidoglycan has been demonstrated to possess significant arthritogenic properties. Intra-articular injection of purified peptidoglycan induces arthritis in a dose-dependent manner, with a single injection resulting in substantial infiltration, primarily of macrophages and polymorphonuclear cells, occasionally leading to bone destruction (Liu et al., 2001). This research was conducted before the arthritogenic properties of lipoproteins were unveiled. In fact, purifying peptidoglycan without *S. aureus* lipoprotein contamination was challenging (Nguyen and Gotz, 2016). It is plausible that the previously demonstrated potent arthritogenic nature of peptidoglycan in that study might have been attributed to lipoprotein contamination. This is further supported by the fact that purified peptidoglycan from an *S. aureus* strain deficient in prelipoprotein lipidation failed to induce macroscopic arthritis in mice (Mohammad et al., 2019). *Staphylococcus aureus* are resistant to lysozyme due to its peptidoglycan modification by O-acetylation of N-acetyl muramic acid. In a local septic arthritis model, a *S. aureus* strain deficient in peptidoglycan o-acetyltransferase, which is highly sensitive to lysozyme secreted by phagocytes, exhibited attenuated virulence in inducing septic arthritis. This suggests that peptidoglycan may not be the primary arthritogenic bacterial component, especially in comparison to lipoproteins. However, it might play a role in resisting host immune killing during development of septic arthritis (Baranwal et al., 2017). In addition to lipoproteins, it has been demonstrated that bacterial DNA containing CpG motifs can also trigger microscopic arthritis, which relies on monocytes and TNF (Deng et al., 1999).

### Regulation system

Virulence factors are under the control of various global regulatory genes, including agr and sarA. The study clearly demonstrated the involvement of the accessory gene regulator (agr) in septic arthritis. This was achieved by utilizing an agr mutant with significantly reduced arthritogenic properties compared to its parental strain (Abdelnour et al., 1993). Notably, when a sar mutant *S. aureus* strain was used, it caused significantly less severe septic arthritis in NMRI mice compared to its parental strain (Nilsson et al., 1996). MgrA belongs to the SarA subfamily. When mice were inoculated with an mgrA mutant strain, they exhibited significantly less severe arthritis, improved weight development, and lower mortality rates compared to those inoculated with parental strains. This suggests that MgrA plays a crucial role in regulating key virulence factors that are important for the development of septic arthritis (Jonsson et al., 2008). Sigma factor B activity controls the activation of regulatory genes such as agr and sarA. rsbU gene is required for posttranslational activation of Sigma factor B. Intravenous inoculation of rsbU-deficient *S. aureus* strain resulted in less severe septic arthritis in mice compared to the strain expressing rsbU, suggesting RsbU regulate vital virulence factors and contribute to the septic arthritis development (Jonsson et al., 2004). Importantly, inactivation of the staphylococcal Agr quorum-sensing system is known to enhance biofilm formation, increasing antibiotic resistance (He et al., 2019). A recent study demonstrates that inappropriate antibiotic treatment can exacerbate prosthetic joint infection in mouse model by promoting quorum cheating and the development of biofilms (He et al., 2023).

### Other virulence factors having impact on septic arthritis

*Staphylococcus aureus* produces a soluble enzyme, staphylokinase, which activates host plasminogen, initiating the fibrinolytic pathway and resulting in the cleavage of fibrin molecules into fibrin degradation products (Bokarewa et al., 2006). Given the species-specific activity of staphylokinase, studies have utilized human plasminogen transgenic mice. Overall, plasminogen activation by *S. aureus*-derived staphylokinase is more protective than pathogenic. In experiments involving human plasminogen transgenic mice inoculated with staphylokinase-expressing strains, there was a significant decrease in mortality, less weight loss, and lower bacterial loads in the kidneys compared to wild-type mice. However, no differences were noted in the severity of septic arthritis (Kwiecinski et al., 2010). Notably, staphylokinase expression by *S. aureus* induces the detachment of mature biofilms. This effect is contingent on plasminogen activation by staphylokinase, suggesting that staphylokinase may function as an anti-virulence factor, particularly in biofilm-related infections such as prosthetic joint infections (Kwiecinski et al., 2016). The role of exoproteases from *S. aureus* in septic arthritis was investigated by utilizing mutants lacking aureolysin, serine protease, and cystine protease. Inactivating the genes encoding these exoproteases did not impact the development of septic arthritis, suggesting a limited role of proteases in the pathogenesis of septic arthritis (Calander et al., 2004).

The Staphylococcal polysaccharide microcapsule has been identified as a critical virulence factor in the development of septic arthritis. This capsule serves to protect the bacteria from engulfment by phagocytes such as neutrophils and macrophages, contributing to the pathogenesis of the condition (Nilsson et al., 1997). Regarding the *S. aureus* toxins, it has been shown that alpha and gamma toxins jointly promote the *S. aureus* virulence in septic arthritis (Nilsson I. M. et al., 1999). TSST-1, a toxin implicated in food poisoning and toxic shock syndrome, was shown to contribute to the arthritogenicity of *S. aureus*, as *S. aureus* deficient in TSST-1 expression led to reduced severity and frequency of septic arthritis in mouse model (Abdelnour et al., 1994b). However, direct injection of TSST-1 in mouse knees did not induce any joint inflammation (Mohammad et al., 2019). By using this model, we have recently revealed that phenol-soluble modulin (PSM) alpha and beta play distinct roles in septic arthritis: PSMα aggravates systemic infection, whereas PSMβ protects arthritis development. Interestingly, we also found that PSMβ has the ability to reduce the neutrophil activating effect of PSMα (Hu et al., 2022; Schultz et al., 2022).

In our model system, a reduced severity and frequency of septic arthritis were observed in *S. aureus* deficient in formylated peptides. This deficiency led to a downregulation in neutrophil recruitment into infected organs, including kidneys and synovial tissues. These findings suggest that formylated peptides serve as virulence factors that play a pivotal role in mediating neutrophil recruitment in septic arthritis (Gjertsson et al., 2012).

## To study the role of host factors in septic arthritis

Host factors, encompassing both innate and adaptive immunity, wield a significant influence in the development of septic arthritis. Table 1 succinctly outlines the discoveries pertaining to the pathogenic or protective roles of these host factors in septic arthritis, bacterial clearance, and lethal sepsis, as observed in mouse models.

**Table 1:** The role of host factors in septic arthritis, bacterial clearance, and lethal sepsis.

Immune system	Septic arthritis	Bacterial clearance	Lethal sepsis	References
Innate immunity				
Neutrophils	−	−	−	Verdrengh and Tarkowski (1997)
Monocytes	+	−	−	Verdrengh and Tarkowski (2000)
NK cells	−	+/−	−	Nilsson M. et al. (1999)
C3	−	−	−	Sakiniene et al. (1999a); Na et al. (2016)
fB	+/−	+/−	+/−	Na et al. (2016)
CR1	−	+/−	+	Sakiniene et al. (1999b)
C3aR	+/−	+/−	+/−	Na et al. (2016)
Nitric oxide	−	−	−	Sakiniene et al. (1997)
Adaptive immunity				
CD4 T cells	+	NA.	+	Abdelnour et al. (1994a)
CD 8 T cells	+/−	+/−	+/−	Abdelnour et al. (1994a)
B cells	+/−	+/−	+/−	Gjertsson et al. (2000)
Tregs	−	+/−	+/−	Bergmann et al. (2020)
				
Cytokines				
TNF α	+	−	−	Hultgren et al. (1998a)
INF γ	+	−	−	Zhao and Tarkowski (1995); Zhao et al. (1998)
IL1	−	+/−	−	Hultgren et al. (2002); Ali et al. (2015a)
IL2	−	−	+/−	Bergmann et al. (2020)
IL4	+	−	−	Hultgren et al. (1998b); Fei et al. (2011)
IL10	−	−	+/−	Gjertsson et al. (2002)
IL17A	−	−	+/−	Henningsson et al. (2010)
IL12	+/−	−	−	Hultgren et al. (2001)
GM-CSF	+/−	+/−	+/−	Verdrengh and Tarkowski (1998)
Other host factors				
MMP7	+	−	+/−	Gjertsson et al. (2005)
MMP9	−	−	−	Calander et al. (2006)
P-selectin	+	−	+/−	Verdrengh et al. (2000)
Vitamin A	−	−	N/A	Wiedermann et al. (1996)
ICAM-1	+	+/−	−	Verdrengh et al. (1996)
IAP	+	−	+/−	Verdrengh et al. (1999)
RAGE	+/−	−	+/−	Mohammad et al. (2016)

NK cells, Nature killer cells; C3, Complement factor 3; fB, Complement factor B; CR1, Complement receptor 1; C3aR, Complement component 3a receptor; Tregs, Regulatory T cells; TNF α, Tumor necrosis factor α, IFN γ, Interferon γ; IL, Interleukin; GM-CSF, Granulocyte-macrophage colony-stimulating factor; MMP, Matrix metalloproteinase; ICAM-1, Intercellular adhesion molecule 1; IAP, Integrin-associated protein; and RAGE, Receptor for advanced glycation end products. +, Pathogenic role in septic arthritis; +/−, Neutral role in septic arthritis; −, Protective role in septic arthritis; N/A, Not assessed. All conclusions were derived from data obtained in the mouse model for hematogenous septic arthritis.

### Innate immunity

It is logical to hypothesize that innate immunity plays a very important role in septic arthritis. This is evident as the increased survival of bacteria in the bloodstream enhances the likelihood of these bacteria ultimately reaching distal joints and causing septic arthritis. Not surprisingly, depletion of neutrophils by anti-ly6G antibodies in mice caused the drastically increased mortality and high susceptibility to *S. aureus* septic arthritis (Verdrengh and Tarkowski, 1997). The intraarticular injection of taurine chloramine, a byproduct of activated neutrophils, resulted in a similar clinical severity of arthritis but caused less extensive damage to the bone and cartilage in the infected joint in the hematogenous septic arthritis model (Verdrengh and Tarkowski, 2005). Sakiniene et al. (1999a) employed cobra venom factor, inducing continuous and excessive activation of C3, which led to the depletion of complement components in mice. This complement depletion system was then applied to a septic arthritis model. They found that complement depletion aggravates *S. aureus* sepsis and septic arthritis (Sakiniene et al., 1999a). The importance of complement system in septic arthritis was proven again in later study where mice lacking the complement component 3 (C3), complement factor B (fB), and receptor for C3-derived anaphylatoxin C3a (C3aR) were used (Na et al., 2016). The results strongly suggest that C3 but not factor B or C2aR deficiency increases susceptibility to hematogenous *S. aureus* septic arthritis, conceivably due to diminished opsonization and phagocytosis of *S. aureus* (Na et al., 2016). In a separate investigation, Sakiniene et al. (1999b) utilized antibodies targeting complement receptor 1 (CR1) to inhibit its activity in a mouse model of hematogenous septic arthritis. Mice treated with these blocking antibodies exhibited a higher incidence and increased severity of septic arthritis, implying that CR1 plays a protective role in this context (Sakiniene et al., 1999b). To understand the role of nitric oxide synthase in septic arthritis, NOS inhibitors (N^G^-monomethyl-l-arginine or N^ω^-nitro-l-arginine methyl ester) were used in the mice model. Interestingly, mice treated with NOS inhibitors displayed increased susceptibility to septic arthritis and this is possibly due to impairment of the intracellular bacteria killing capacity of macrophages (Sakiniene et al., 1997). The involvement of natural killer (NK) cells in septic arthritis was investigated by depleting NK1.1+ cells in mice using anti-PK136 antibodies. NK cell-depleted mice exhibited a significantly higher incidence and severity of septic arthritis, although bacterial clearance *in vivo* remained similar (Nilsson N. et al., 1999).

Interestingly, not all cells in innate immunity play a protective role in septic arthritis. Monocytes and macrophages appear to contribute to the development of septic arthritis pathologically. This is evident from experiments where the depletion of monocytes using etoposide treatment in mice led to less severe septic arthritis, despite an increase in mortality among the monocyte-depleted mice (Verdrengh and Tarkowski, 2000).

### Adaptive immunity

To elucidate the role of B cells in septic arthritis, gene-targeted B-cell-deficient μMT mice were inoculated with the *S. aureus* LS-1 strain, and the severity of septic arthritis was compared between the experimental and control groups. No significant difference was observed between the mature B cell-depleted mice and the wild-type controls. This suggests a limited role of B cells in septic arthritis, at least in individuals without prior *S. aureus* infections (Gjertsson et al., 2000). Similar data were shown when CD22 deficient mice were used (Gjertsson et al., 2004).

The study revealed that mice deficient in MHC class II exhibited a reduced occurrence of septic arthritis, underscoring the essential role of MHC class II expression in the development of this condition (Abdelnour et al., 1997). The significance of CD4 T cells and V beta 11+ T cells in the development of septic arthritis has been established. Pretreatment of mice with anti-CD4 or anti-V beta 11 antibodies inhibited the development of arthritis when intravenously inoculated with *S. aureus*, whereas anti CD8 had no effect on septic arthritis development. This suggests that CD4 T cells and V beta 11+ T cells play a crucial role in the pathogenesis of septic arthritis in this experimental setting (Abdelnour et al., 1994a). Furthermore, our recent findings indicate that treatment with CTLA4 Ig, which inhibits T cell activation, significantly amplifies the severity of septic arthritis. This underscores the complex involvement of T cell activation in the regulation of septic arthritis (Ali et al., 2015b). The use of anti-CD25 antibodies to deplete Tregs in both NMRI and C57/BL6 mice did not notably impact weight loss or bacterial clearance. However, it resulted in a more severe clinical septic arthritis, indicating the protective function of Tregs in this context. This observation was reinforced by the finding that low-dose IL2 treatment expanded the Treg compartment and mitigated the severity of septic arthritis (Bergmann et al., 2020).

### Cytokines

Transcription factors regulating pro-inflammatory cytokines have been studied. Nuclear factor kappa B (NF-kappa B) and activator proteins (AP-1) play pivotal roles as transcription factors in the induction of numerous inflammatory genes. Antisense therapies targeting NF-kappa B or AP-1 resulted in heightened severity of septic arthritis lesions and an increased bacterial load in the kidneys associated with septic arthritis (Gjertsson et al., 2001). Mice lacking the T-box transcription factor (T-bet) exhibited heightened severity of septic arthritis in the early phase of the disease, along with increased kidney bacterial load and greater weight loss (Gjertsson et al., 2001). The Janus kinase (JAK) family comprises intracellular, non-receptor tyrosine kinases involved in transmitting cytokine-mediated signals through the JAK–STAT pathway. While tofacitinib, a JAK inhibitor, was demonstrated to enhance susceptibility to septic arthritis in mice, it exhibited a positive impact on the survival of *S. aureus*-induced sepsis (Jarneborn et al., 2020).

In investigating the role of IFN-gamma in septic arthritis, mice lacking the IFN-gamma receptor were employed. These mice exhibited a higher incidence and increased severity of septic arthritis, coupled with elevated mortality rates compared to wild-type mice (Zhao and Tarkowski, 1995). The later study showed similar results using administration of both exogenous IFN-gamma and anti-IFN-gamma antibodies. The results showed that IFN-gamma treatment decreased mortality but increased the incidence of septic arthritis. Conversely, the use of anti-IFN-gamma antibodies alleviated septic arthritis symptoms but raised mortality rates. These findings indicate that while IFN-gamma expression is pathogenic for septic arthritis, it plays a protective role in preventing lethal sepsis (Zhao et al., 1998). The study demonstrated that mice lacking the interleukin-1 receptor (IL-1 receptor knockout mice) exhibited significantly higher mortality rates and more severe septic arthritis compared to wild-type mice. These findings suggest the crucial role of signaling through the IL-1 receptor A in the development of septic arthritis (Hultgren et al., 2002). Consistent with these findings, a subsequent study revealed that treatment with IL-1 blockade resulted in an increased severity of septic arthritis in the mouse model, further supporting the notion that IL-1 plays a protective or mitigating role in this context (Ali et al., 2015a). The administration of a low-dose IL-2 treatment through intraperitoneal injection of a recombinant adeno-associated virus vector effectively mitigated the severity of septic arthritis. This intervention also maintained the host’s capacity to clear the infection, indicating a protective role of IL-2 in septic arthritis (Bergmann et al., 2020). Deficiency in IL-4 results in a notably reduced incidence of septic arthritis, along with a decreased bacterial load in the joints and kidneys. This phenomenon is explained by *in vitro* findings indicating that IL-4 inhibits the intracellular killing of *S. aureus* by macrophages (Hultgren et al., 1998b). Mice lacking both TNFα and β demonstrated less severe *S. aureus* septic arthritis, yet exhibited increased mortality rates. This paradoxical outcome was accompanied by reduced bacterial clearance compared to wild-type controls. These findings imply that TNF plays a dual role, acting as a crucial inflammatory mediator in the response to septic arthritis, while concurrently serving as a protective cytokine in lethal sepsis (Hultgren et al., 1998a). Importantly, our later study demonstrated that antibiotics combined with TNF inhibitor (enteracept) indeed is superior than antibiotics alone in treatment of septic arthritis in our mouse models (Fei et al., 2011). Mice deficient in IL-10 developed more severe septic arthritis, characterized by a higher bacterial load in the kidneys compared to the wild-type mouse strains. This suggests a protective role of IL-10 in the context of septic arthritis (Gjertsson et al., 2002). IL-17A knockout mice exhibited more severe septic arthritis lesions, increased weight loss, and higher bacterial load in kidneys, indicating a protective role of IL-17A in septic arthritis (Henningsson et al., 2010).

Certain cytokines, such as IL-12, were found to play no significant role in septic arthritis. While there was no discernible difference in severity of septic arthritis in mice lacking IL-12, there was a notable increase in mortality. This suggests that IL-12 may not directly influence the development or severity of septic arthritis in this particular context (Hultgren et al., 2001). Pretreatment of mice with granulocyte-macrophage colony-stimulating factor (GM-CSF) had no discernible impact on the development of septic arthritis, despite an increase in the total number of leukocytes and the granulocyte fraction. This suggests that the augmentation of leukocytes and granulocytes alone induced by GM-CSF does not influence the progression of septic arthritis (Verdrengh and Tarkowski, 1998).

### Other host factors

Matrix metalloproteinases (MMPs) form a family of structurally related endopeptidases crucial for the normal upkeep of the extracellular matrix. Intriguingly, the absence of MMP9 leads to significantly more severe septic arthritis, characterized by elevated bacterial loads in both the joints and kidneys (Calander et al., 2006). In contrast, mice deficient in MMP-7, when inoculated with *S. aureus*, exhibited less severe septic arthritis. However, they displayed higher bacterial loads in the kidneys, suggesting a pathogenic role of MMP7 in septic arthritis (Gjertsson et al., 2005).

In order to investigate the role of selectins in septic arthritis, mice lacking P-selectin and mice treated with fucoidan, a carbohydrate molecule that inhibits selectin functions, were intravenously injected with *S. aureus*. Interestingly, the blockade or deficiency of selectins resulted in less severe septic arthritis during the initial phase of the disease. However, it was also associated with higher bacterial loads in the kidneys (Verdrengh et al., 2000).

The coagulation and fibrinolytic system may play a role in the pathogenesis of septic arthritis. During bacterial infections, there is a shift in hemostatic balance toward coagulation. This is attributed to the increased synthesis of tissue factor, a catalyst for the coagulation protease cascade, the inhibition of the anticoagulant pathway, and the impairment of fibrinolysis through the upregulation of plasminogen activator inhibitor type-1, a significant inhibitor of tissue-type and urokinase plasminogen activators (Levi et al., 2004). In a hematogenous septic arthritis model, mice treated with low molecular weight heparin, an anticoagulant, experienced more frequent and severe septic arthritis compared to controls (Sakiniene and Tarkowski, 2002). On the contrary, treatment with tranexamic acid, a fibrinolysis blocker, resulted in higher mortality and more severe septic arthritis (Klak et al., 2010). These results may suggest the importance of hemostatic balance of coagulation/fibrinolysis in protecting septic arthritis development.

In a study using a rat model for septic arthritis, the impact of vitamin A deficiency was examined. Interestingly, vitamin A deficient rats displayed an increased susceptibility to *S. aureus* septic arthritis. This heightened susceptibility could be attributed to factors such as T cell hyperactivity, impaired phagocyte function, and decreased complement activity (Wiedermann et al., 1996).

Several lines of evidence indicate that leukocyte-endothelial cell adhesion and leukocyte extravasation likely contribute to septic arthritis. Deficiency in intercellular adhesion molecule 1 (ICAM-1) or treatment with anti-ICAM-1 antibodies resulted in a less severe form of septic arthritis but increased mortality. This highlights the dual role of ICAM-1 in septic arthritis: while it provides protection against lethal sepsis, it concurrently exacerbates the development of septic arthritis (Verdrengh et al., 1996). In order to investigate the role of P-selectins in septic arthritis, mice lacking P-selectin and mice treated with fucoidan, a carbohydrate molecule that inhibits selectin functions, were intravenously injected with *S. aureus*. Interestingly, the blockade or deficiency of selectins resulted in less severe septic arthritis during the initial phase of the disease. However, it was also associated with higher bacterial loads in the kidneys (Verdrengh et al., 2000). Integrin-associated protein (IAP) serves as a receptor for thrombospondin family members and regulates various cellular activities such as leukocyte adhesion, migration, and phagocytosis (Brown and Frazier, 2001). The absence of IAP in mice conferred resistance to the development of septic arthritis, underscoring the importance of leukocyte adhesion and migration into the joints in the progression of septic arthritis (Verdrengh et al., 1999). In investigating the role of the receptor for advanced glycation end products (RAGE) in septic arthritis, the use of RAGE-deficient mice revealed no significant differences in the severity of septic arthritis or mortality rate, indicating a limited role for RAGE in the context of septic arthritis (Mohammad et al., 2016).

Advanced age has been recognized as a significant risk factor for septic arthritis in patients (Kaandorp et al., 1995). Nonetheless, our recent study, employing aging and TLR-2 deficient mice, disclosed that increased age stood out as the primary contributor to heightened mortality rates and changes in spleen weight (Hu et al., 2023). Surprisingly, the severity of septic arthritis remained unaltered by aging, even though aged mice demonstrated an insufficient antibody response to *S. aureus* infection (Gupta et al., 2023).

## To study the novel therapeutic strategies

While there has been some progress in the development of new treatments for septic arthritis, it is noteworthy that the current treatment options have remained largely unchanged for the past 3 decades.

### Vaccine development

Developing a vaccine against *S. aureus* presents significant challenges, with numerous clinical trials falling short in terms of efficacy in patients (Miller et al., 2020; Proctor, 2023). *Staphylococcus aureus* surface proteins play the vital role in induction of septic arthritis, as deficiency in Sortas A, ClfA, and collagen adhesin in *S. aureus* greatly reduced the bacterial arthritogenic capacity. Not surprisingly, the vaccination strategies against those molecules have been tried in the hematogenous septic arthritis models. It has been shown that both passive and active immunization against ClfA protect the septic arthritis development in mice (Josefsson et al., 2001). Also, vaccination with a recombinant version of the collagen adhesin protect the mice against a heterologous challenge of CNA1 *S. aureus* (Nilsson et al., 1998). In the past decade, there has been a significant focus on developing vaccine cocktails in humans that target multiple key virulence factors in *S. aureus* (Clegg et al., 2021). One notable vaccine formulation involved using aluminum hydroxide as an adjuvant and included five *S. aureus* antigens in what was referred to as the four-component *S. aureus* vaccine (4C-Staph). These antigens comprised a genetically detoxified version of the secreted alpha-toxin hemolysin (Hla), two surface-exposed antigens (FhuD2 and Csa1A), and EsxAB, a fusion protein of two secreted proteins, EsxA and EsxB. This 4C-Staph vaccine formulation demonstrated efficacy in protecting mice from various *S. aureus* infections, including abscesses, peritonitis, pneumonia, and skin infections (Bagnoli et al., 2015). Furthermore, in a systemic septic arthritis model, vaccination with 4C-Staph exhibited a protective effect, leading to reduced bacterial colony-forming units (CFU) in joints and kidneys (Corrado et al., 2016). Recently, SA4Ag has been developed. SA4Ag comprises capsular polysaccharide types 5 and 8 CRM197 conjugates, a mutant form of clumping factor A (Y338A-ClfA), and the manganese transporter subunit C (MntC). Notably, SA4Ag has demonstrated impressive results, significantly reducing bacterial load in deep tissue infections, bacteremia, pyelonephritis models, and even completely preventing infectious endocarditis in a rat model (Scully et al., 2021). However, it is important to note that this vaccine did not prove efficacious in preventing *S. aureus* infections following spinal surgery in patients (Hassanzadeh et al., 2023). One potential explanation for the failure of the *S. aureus* vaccine could be the excessive production of non-protective antibodies characterized by an increased α2,3 sialylation pattern. These antibodies have impeded opsonophagocytosis capacity and engage in direct competition with protective antibodies in individuals who have previously experienced *S. aureus* infections (Tsai et al., 2022). It is well known that a significant portion of the population is colonized by *S. aureus* (Sakr et al., 2018), and this colonization likely represents a prior interaction between the host and *S. aureus*.

Given the above reason and the potential immunodeficiency observed in susceptible patient populations, passive immunization emerges as a promising alternative. The autolysin enzyme plays a vital role in both cell wall biosynthesis and degradation during binary fission, with the Glucosaminidase (Gmd) subunit identified as an immunodominant antigen (Varrone et al., 2011). Circulating anti-Gmd antibodies serve as a serum biomarker indicating protective immunity against *S. aureus* in patients with orthopedic infections (Kates et al., 2020). In a murine model of implant-associated osteomyelitis, passive immunization with anti-Gmd monoclonal antibodies was tested. The results demonstrated a significant reduction in infection severity, assessed through bioluminescent imaging of bacteria, micro-CT evaluation of osteolysis, and histomorphometry of abscess numbers (Varrone et al., 2014). Indeed, these findings suggest the potential efficacy of passive immunization in the treatment of septic arthritis.

### Inhibition of exaggerated immune response

The underlying hypothesis, held for many years, attributes the primary cause of bone degradation and lasting joint dysfunction in septic arthritis to an exaggerated immune response. To mitigate this immune response and minimize the risk of enduring joint damage, Professor Tarkowski and colleagues proposed a combined approach involving antibiotics and immunomodulatory corticosteroid therapy, as demonstrated in the hematogenous septic arthritis model (Sakiniene et al., 1996). Subsequently, two clinical trials showcased potential benefits of this combination treatment for pediatric septic arthritis patients (Odio et al., 2003; Harel et al., 2011). Nonetheless, due to the limitations of these clinical trials in terms of quality, the adoption of combination therapies has not yet been endorsed in clinical practice. There remains a need for further randomized studies to ascertain their effectiveness. Interestingly, it is noteworthy that early administration of non-steroidal anti-inflammatory drugs (NSAIDs) in combination with appropriate systemic antibiotics has demonstrated a beneficial effect in protecting articular cartilage from damage in a local septic arthritis rabbit model (Tahami et al., 2020). This suggests that the inhibition of cyclooxygenase, without a profound impact on immune responses, may have a joint-protecting effect beyond pain relief alone.

Given the pivotal role of TNF in septic arthritis development (Hultgren et al., 1998a), we evaluated the combination of antibiotics and TNF inhibitors using our hematogenous model. Our findings indicate that this combined treatment is superior than antibiotics alone, evidenced by improvements in both clinical severity and bone erosion as observed in histopathology (Fei et al., 2011). However, recent data from the similar model system highlight potential risks associated with such combination therapies, particularly in light of the ongoing challenge of antibiotic resistance (Ali et al., 2015a,b), which emphasizes the necessity for novel therapies employing alternative mechanisms to address septic arthritis effectively.

### Blocking osteoclast activation

Focal bone destruction in autoimmune arthritis is partially due to excess bone resorption by osteoclast activation, which is mediated by increased local expression of RANKL relative to the expression of its decoy receptor osteoprotegerin (OPG; Walsh and Gravallese, 2010). Osteoclasts not only exist inside the bone, but can also be derived from mature monocytes and macrophages when a suitable microenvironment is provided (Udagawa et al., 1990). Monocytes/macrophages have been shown to mediate bone erosions in the arthritis induced by other *S. aureus* components, such as bacterial DNA (Deng et al., 1999) and antibiotic-killed *S. aureus* (Ali et al., 2015c). It has been shown before that *S. aureus* enhances bone resorption and periosteal osteoclast formation by increasing osteoblast RANKL production through TLR2 in an *ex vivo* setting (Kassem et al., 2016). Treatment with bisphosphonates in combination with antibiotics and corticosteroids significantly reduced the activity of osteoclasts and reduced the risk of skeletal destruction (Verdrengh et al., 2007). Our preliminary data demonstrate that both monocyte depletion and a RANKL inhibitor fully prevent the joint destruction in septic arthritis, strongly suggesting that activated osteoclastogenesis is the main cause of joint damage in septic arthritis and targeting the key molecules in osteoclast activation may improve dramatically the treatment for joint damage in patients with septic arthritis.

Patients with septic arthritis may experience additional benefits, such as improved outcomes in osteoporosis, through anti-RANKL treatment. It is evident that rapid systemic bone resorption occurs during *S. aureus* septic arthritis (Verdrengh et al., 2006). Recent studies propose that this bone resorption effect may be induced by *S. aureus* lipoproteins, specifically through their lipid moiety acting on monocytes/macrophages (Schultz et al., 2022). Given that advanced age is a major risk factor for septic arthritis and osteoporosis is common in elderly individuals, older patients with septic arthritis may face an elevated risk of severe osteoporosis, particularly in the affected joints. Anti-RANKL treatment presents a potential avenue to reverse this process and mitigate the risk of fractures.

### Other treatments

Treatment failure in septic arthritis has been linked to free-floating, antibiotic-resistant *S. aureus* biofilm aggregates formed in synovial fluid (Gilbertie et al., 2019). Host-derived fibrin has been identified as a major component of the *S. aureus* biofilm matrix (Kwiecinski et al., 2016). The dispersal of synovial fluid biofilm aggregates by tissue plasminogen activator has been shown to restore antimicrobial activity both *in vitro* (Gilbertie et al., 2019) and *in vivo* (Kwiecinski et al., 2015).

Intriguingly, the evaluation of antibiotics combined with ultrasound-triggered microbubble destruction on *S. aureus* aggregates in a porcine intra-articular infection model demonstrated an increase in antibiotic activity. This suggests that this combination treatment may serve as an important adjunct for the treatment of septic arthritis (Zhao et al., 2023).

Mesenchymal stromal cells (MSC), known for their antimicrobial and immunomodulatory properties, were assessed in combination with antibiotics in an equine model for septic arthritis. The study revealed beneficial effects in pain scores, ultrasound results, and proinflammatory markers compared to the antibiotics alone group (Pezzanite et al., 2022). This suggests that activated MSC combined with antibiotics may be a promising approach to manage joint infections with drug-resistant bacteria.

Platelet-rich plasma lysate, despite its unknown active components, demonstrated effectiveness against bacterial biofilm aggregates *in vitro*. Additionally, it exhibited synergism with amikacin against aminoglycoside-tolerant biofilm aggregates (Gilbertie et al., 2020). In an equine model of infectious arthritis, the antibiotics in combination with platelet-rich plasma lysate reduced bacterial concentrations in synovial fluid and synovial tissue, leading to lower systemic and local inflammation compared to antibiotics alone. Importantly, this combination therapy also reduced the loss of infection-associated cartilage proteoglycan content in articular cartilage and decreased synovial tissue fibrosis and inflammation (Gilbertie et al., 2022).

## Concluding remarks

Septic arthritis represents a severe clinical condition with the potential to induce lasting joint dysfunction. The primary pathogen responsible for septic arthritis is *S. aureus*. The mouse model for septic arthritis stands as a powerful tool for enhancing our understanding of the intricate interplay between bacteria and hosts, unraveling disease mechanisms, and facilitating the discovery of novel therapies. However, large animal models, specifically involving pigs and horses, play a crucial role in bridging the translational gap. These models serve as a necessary step, enabling a more realistic evaluation of therapy efficacy before embarking on clinical trials in human patients.

These models have been instrumental in identifying crucial virulence factors integral to septic arthritis. Notably, *S. aureus* surface proteins such as vWbp, clumping factor A, and protein A play pivotal roles in facilitating bacterial invasion into the joint cavity. Conversely, joint inflammation and subsequent destruction are predominantly caused by Staphylococcal lipoproteins.

On the host front, innate immunity assumes a crucial role in eradicating bacteria in the blood stream before they invade the joint cavity. Neutrophils and the complement system, notably C3, are of paramount importance in this process. Interestingly, while monocytes and macrophages appear implicated in provoking joint inflammation and erosion, they maintain a pivotal role in eliminating bacteria. In the context of the adaptive immune system, the roles of B and T cells in septic arthritis, particularly within animal models, remain less distinct. A multitude of cytokines significantly influences the progression of septic arthritis.

Turning to innovative therapies, vaccination directed at virulence factors emerges as a prominent initial choice. However, vaccines targeting *S. aureus* are currently unavailable. The vaccine cocktails targeting several key virulence factors implicated in septic arthritis pathogenesis holds promise. Notably, a combination of antibiotics and interventions inhibiting the recruitment of monocytes to local joints or suppressing osteoclastogenesis activation within affected joints may represent novel therapeutic avenues. Strategies aimed at disrupting bacteria biofilm aggregates in joints have the potential to enhance antibiotic efficacy, ultimately improving the prognosis of septic arthritis. These novel approaches hold promise in preventing joint destruction and subsequent dysfunction associated with the disease.

## Author contributions

TJ: Conceptualization, Data curation, Formal analysis, Funding acquisition, Investigation, Methodology, Project administration, Resources, Software, Supervision, Validation, Visualization, Writing – original draft, Writing – review & editing.

## In memoriam

This review article commemorates the significant contributions of Andrej Tarkowski at Sahlgrenska University Hospital to septic arthritis research. His pioneering work enhanced our understanding of this devastating disease, despite his unfortunate demise at age of 56 due to colon cancer metastasis in 2008.
